# Exogenous Hemin alleviated cadmium stress in maize (*Zea mays* L.) by enhancing leaf photosynthesis, AsA-GSH cycle and polyamine metabolism

**DOI:** 10.3389/fpls.2022.993675

**Published:** 2022-09-08

**Authors:** Lin Piao, Yong Wang, Xiaoming Liu, Guangyan Sun, Shiyu Zhang, Junyao Yan, Yang Chen, Yao Meng, Ming Li, Wanrong Gu

**Affiliations:** ^1^College of Agriculture, Northeast Agricultural University, Harbin, China; ^2^Maize Research Institute, Heilongjiang Academy of Agricultural Sciences, Harbin, China; ^3^Heilongjiang Kenfeng Seed Industry Co., Ltd., Harbin, China; ^4^Heilongjiang Academy of Land Reclamation Sciences, Harbin, China

**Keywords:** Hemin, cadmium stress, photosynthetic capacity, AsA-GSH cycle, polyamine metabolism

## Abstract

Cadmium (Cd) stress is one of the principal abiotic stresses that inhibit maize growth. The research was to explore (hemin chloride) Hemin (100 μmol L^−1^) on photosynthesis, ascorbic acid (AsA)-glutathione (GSH) cycle system, and polyamine metabolism of maize under Cd stress (85 mg L^−1^) using nutrient solution hydroponics, with Tiannong 9 (Cd tolerant) and Fenghe 6 (Cd sensitive) as experimental materials. The results showed that Hemin can increase leaf photosynthetic pigment content and ameliorate the ratio of Chlorophyll a/chlorophyll b (*Chla*/*Chlb*) under Cd stress. The values of ribose 1, 5-diphosphate carboxylase/oxygenase (RuBPcase) and phosphoenolpyruvate carboxylase (PEPCase), and total xanthophyll cycle pool [(violoxanthin (V), antiflavin (A) and zeaxanthin (Z)] increased, which enhancing xanthophyll cycle (DEPS) de-epoxidation, and alleviating stomatal and non-stomatal limitation of leaf photosynthesis. Hemin significantly increased net photosynthetic rate (*P_n_*), stomatal conductance (*g_s_*), transpiration rate (*T_r_*), photochemical quenching coefficient (*qP*), *PSII* maximum photochemical efficiency (*F_v_/F_m_*), and electron transfer rate (*ETR*), which contributed to the improvement of the PSII photosynthetic system. Compared with Cd stress, Hemin can reduce thiobartolic acid reactant (TBARS) content, superoxide anion radical (O_2_^−^) production rate, hydrogen peroxide (H_2_O_2_) accumulation, and the extent of electrolyte leakage (EL); decreased the level of malondialdehyde (MDA) content and increased the activities of superoxide dismutase (SOD), peroxidase (POD) and catalase (CAT); slowed the decrease in dehydroascorbic acid reductase (DHAR) and monodehydroascorbate reductase (MDHAR) activity and the increase in glutathione reductase (GR) and ascorbate peroxidase (APX) activity in leaves; promoted the increase in AsA and GSH content, decreased dehydroascorbic acid (DHA) and oxidized glutathione (GSSG), and increased AsA/DHA and GSH/GSSG ratios under Cd stress. Hemin promoted the increase of conjugated and bound polyamine content, and the conversion process speed of free putrescine (Put) to free spermine (Spm) and spermidine (Spd) in maize; decreased polyamine oxidase (PAO) activity and increased diamine oxidase (DAO), arginine decarboxylase (ADC), ornithine decarboxylase (ODC) and S-adenosylmethionine decarboxylase (SAMDC) enzyme activities in leaves under Cd stress.

## Introduction

Cadmium stress inhibits cell division and elongation, and hinders DNA and RNA expression in plants (Sarker et al., 2002; Wang et al., 2021). Maize has the characteristics of wide distribution and a large area in the planting area. Recently, it has been reported that the heavy metal pollution in the corn production area is serious, and the cadmium content in the maize grain exceeds the standard (Wang et al., 2018; Zhou et al., 2020). At the same time, it has also been reported that the growth and development of maize are seriously inhibited on the cadmium polluted farmland, and the biological yield and grain yield are significantly reduced (Anjum et al., 2015; Guan et al., 2021). Studies have shown that Cd stress reduces the chlorophyll content of maize leaves, which decreases with the increase of Cd concentration. The decrease of chlorophyll content may be due to the combination of Cd and the enzyme protein sulfhydryl groups of various enzymes in the chloroplast, which destroys the structure of chloroplast, disappears the stromal lamellae, disrupts the chloroplast grana stacking, reduces the synthesis of chlorophyll and promotes chlorophyll decomposition (Sun et al., 2012). Cadmium ion has non-redox properties, which can combine with the enzyme active center or protein sulfhydryl group of cells to replace the essential elements Ca^2+^, Mg^2+^, Zn^2+^, etc. to produce free ions, thus causing oxidative stress (Wang et al., 2004; Wu, 2009). The imbalance between the excessive production of reactive oxygen species (ROS) and the antioxidant capacity of plants is an important cause of Cd toxicity (Jiang et al., 2020; Sun et al., 2022). Polyamines, widely found in biological cells, are a class of biologically active low molecular weight aliphatic nitrogenous bases produced during biological metabolism (Liu and Moriguchi, 2007). Polyamines mainly include Put, Spm and Spd, and exist in free, soluble conjugated and insoluble bound forms. Polyamines play a role in stabilizing cell structure and are involved in various essential plant physiological processes (Li et al., 2018).

Plants have specific defense mechanisms against Cd, including changing rhizosphere chemical properties, heavy metal ion regionalization and chelation to reduce Cd toxicity (Gu and Zhou, 2002; Zhao et al., 2022). Previous studies have shown that exogenous nitric oxide (NO) can alleviate the damage of Cd stress on seedling roots and reduce EL (Ahmed et al., 2002). Spermidine and polyamine treatment can alleviate the inhibition of Cd stress on photosynthesis (Sun and Sun, 2019). In recent years, progress has been made in improving crop resistance to Cd stress using melatonin (Fernándezab et al., 2013; Gu et al., 2021; Altaf et al., 2022a), methyl jasmonate (Shan et al., 2016), 5-Aminolevulinic acid (Qin et al., 2006), chitosan (Ding et al., 2007), betaine (Zhao et al., 2001), salicylic acid (Ren et al., 2009), ascorbic acid (Lv et al., 2009) and nanotitania particle (Chen et al., 2015).

Hemin is a purified form of natural heme *in vitro*, which is generally obtained by isolation and purification from animal blood (Liu and Moriguchi, 2007). In recent years, researchers have begun to pay attention to the application of Hemin in horticultural and field crops, focusing on the role (Dingenen et al., 2019) of Hemin in inducing plant resistance to abiotic stresses such as heavy metals, salinity, UV-B, and drought. Studies showed that exogenous Hemin could noticeably mitigate the oxidative damage of Alfalfa caused by mercury stress (Han et al., 2007). Hemin can alleviate root elongation inhibition caused by salt stress (Xie et al., 2011), and alleviate salt stress damage on tobacco seedlings (Liu et al., 2021). After Hemin treatment, wheat seedlings’ salt resistance and rice’s Cd resistance significantly improved (Huang et al., 2006). Hemin can effectively alleviate the damage of Cr^6+^ Stress on wheat (Sun et al., 2020). Previous studies mainly focused on the responses of Cd tolerance among different maize varieties and comparing their Cd tolerance differences. In this study, we discussed the mechanisms of Hemin to enhance Cd tolerance in maize from the perspectives of leaf photosynthesis, antioxidant enzyme system and polyamine metabolism. Our study will provide some theoretical basis for reducing Cd accumulation and Cd toxicity risk in maize, and paving the way for applying Hemin in maize stress-resistance production.

## Materials and methods

### Material and experimental design

The experiment was conducted in Northeast Agricultural University. Hemin was purchased from Sigma Company (CAS number, 16009–13-5; molecular formula, C_34_H_32_ClN_4_O_4_Fe; molecular weight, 651.96). CdCl_2_ was used to simulate Cd stress [CAS number: 10108–64-2; Sigma-Aldrich (Shanghai) Trading Co., Ltd., Shanghai, China].

Based on the preliminary results, Tiannong 9 (Cd tolerant; Fushun Tiannong Seed Co., Ltd., Liaoning, China) and Fenghe 6 (Cd sensitive; Heilongjiang Fenghe Seed Co., Ltd., Heilongjiang, China) were selected. The seeds were disinfected with NaClO (10%) for 10 min, rinsed and soaked for 7 h with distilled water. Afterwards, the seeds were placed in trays and dark germinated in a constant temperature incubator for 48 h. Two-leaf-stage seedlings were selected and set in 1/2-strength Hoagland nutrient solution (40 L, pH 6.8). The solution was changed every 3 days, and the air pump was timed to ventilate (40 min h^−1^), with room light for 12 h and dark for 12 h. The treatments were set as follows: (1) 1/2-strength Hoagland nutrient solution (CK); (2) 1/2-strength Hoagland nutrient solution +100 μmol L^−1^ Hemin; (3) 1/2-strength Hoagland nutrient solution +85 mg L^−1^ CdCl_2_; (4) 1/2-strength Hoagland nutrient solution +85 mg L^−1^ CdCl_2_ + 100 μmol L^−1^ Hemin. After treatment with 85 mg L^−1^ CdCl_2_ for 24 h, Hemin was added to the nutrient solution to reach a concentration of 100 μmol L^−1^. pH was adjusted once a day, and incubator temperature was 25°C, light intensity was 400 μmol m^−2^ s^−1^, and relative humidity was 60–70%.

## Measurement and methods

### Analysis of leaves photosynthetic parameters and key enzyme activities

Chlorophyll a (*Chla*), chlorophyll b (*Chlb*) and carotenoid (*Car*) contents: 0.5 g leaf sample was ground into a homogenate by adding acetone (80% V/V, 5 ml) in a mortar. The samples were centrifuged at 10000 g, 4°C for 10 min and the absorbance was measured by spectrophotometer (UV-5500, Shanghai Chemical Laboratory Equipment Co., Ltd., Beijing, China) at wavelengths 470, 646 and 663 nm. The content of *Chla*, *Chlb and Car* were calculated according to Arnon’s equation (Li et al., 2018).

Lutein cycle components (V, A, Z): 0.5 g leaf sample was soaked in liquid nitrogen and ground into a homogenate with a small amount of Na_2_CO_3_. The lutein pigment in the leaves were extracted with 100% acetone for 1 min under dark conditions. Then the extracts were centrifuged at 2500 g, 4°C for 10 min, and the supernatant was filtered through a 0.2 μm syringe filter. V, A, Z were analyzed with 25 μl of the filtrate using a Shimadzu LC-20A high performance liquid chromatograph (LC-20A, Shanghai Zhiyan Scientific Instrument Co., Ltd., Shanghai, China; Li et al., 2006). DEPS value was calculated by (A + Z)/(V + A + Z).

Gas exchange parameters: Using a LI-6400 portable photosynthesis system (LI-COR Inc., United States), the photosynthetic parameters such as *P_n_*, *C_i_*, *g_s_*, and *T_r_* were measured in fully expanded inverted triple leaves in a greenhouse at an atmospheric CO_2_ concentration of 400 μmol mol^−1^, a temperature of 25°C, and a light intensity of about 400 μmol m^−2^ s^−1^. Leaf stomatal limitation (*L_s_*) was calculated according to the equation *L_s_* = 1-*C_i_*/*C_a_*, where *Ca* is the ambient CO_2_ concentration, and water use efficiency (WUE) is calculated according to the equation WUE = *P_n_*/*T_r_*.

Chlorophyll fluorescence parameters: Chlorophyll fluorescence was measured using a PAM-2100 chlorophyll fluorometer (Walz, Germany). After dark adaptation for 30 min, minimum fluorescence (*F_o_*) and maximum fluorescence (*F_m_*) was obtained by irradiating the measured light (<0.05 mm m^−2^ s^−1^) and saturated pulsed light (8,000 μm m^−2^ s^−1^), respectively. The photosynthetic steady-state fluorescence (*F_s_*) was measured by turning on the action light (300 μm m^−2^ s^−1^), the maximum fluorescence (*F_m_′*) was obtained by turning on the saturated pulsed light (8,000 μm m^−2^ s^−1^) again, and the minimum fluorescence under light (*F_o_*’) was obtained by turning off the action light and turning on the far-red light immediately. Other parameters were valued as follows: *F_v_*/*F_m_* = (*F_m_−F_o_*)/*F_m_*; *ΦPSII* = (*F_m_′−F_s_*)/ *F_m_*′; *ETR* = (*ФPSII* × 0.5 × PPFD × 0.84), where PPFD is the light flux density; *qP* = (*F_m_*′*−F_s_*)/(*F_m_*′*−F_o_’*); and non-photochemical quenching coefficient (*NPQ*) = (*F_m_−F_m_′*)/*F_m_*′.

RUBPCase and PEPCase activities: 0.5 g fresh leaf sample was extracted by grinding with a small amount of quartz sand and 3 ml of pre-cooled extraction solution which contained 100 mmol L^−1^ Tris–HCl (pH 7.8), 10 mmol L^−1^ MgCl_2_, 1 mmol L^−1^ EDTA, 20 mmol L^−1^ β-mercaptoethanol, 10% (W/V) glycerin and 1% PVP. After filtering, the filtrate was centrifuged at 4°C, 15000 rpm for 10 min, and the supernatant was used for enzyme activity determination. PEPCase and RuBPCase activity were calculated by reference to the enzyme coupling method (Roh and Choi, 2006; Shahid et al., 2016).

### Determination of active oxygen metabolism and relevant indicators of antioxidant system

TBARS content: 0.5 g leaf tissue was ground with phosphate buffer (3 ml, pH 7.0) and centrifuged at 20000 g for 20 min. 1 ml of supernatant was mixed well with 4 ml of 0.5% TBA (thiobarbituric acid), reacted at 95°C for 30 min and then cooled rapidly. After that, it was centrifuged at 10000 g for 10 min. Its A532 and non-specific A600 was read in supernatant, and the TBARS content was expressed as μmol g^−1^ FW (Wang et al., 2014).

O_2_^−^· production rate and H_2_O_2_ content: 100 μl chloroplast supernatant was added to ice-cold PBS buffer (200 μl, 65 mm, pH 7.8) and hydroxylamine hydrate chloride (300 μl), placed at 30°C for 20 min, and then centrifuged 3,000 g for 5 min at room temperature. The extract (300 μl) was added to the tube with sulfonamide (500 μl, 17 mm) and α-naphthylamine (500 μl, 7 mm). The mixture was then left at 30°C for 20 min and then mixed with pure ether (2.25 ml). The absorbance was measured at 530 nm and the O_2_^−^· production rate was calculated from the NaNO_2_ standard curve. To determine H_2_O_2_ content, leaf tissue (0.5 g) was ground into a homogenate in phosphate buffer (3.0 ml, 50 mm, pH 6.8). Then it was centrifuged at 6000 g for 25 min, and 3 ml of the extract was mixed with 0.1% titanium chloride in 20% sulfuric acid. Afterward, the mixture was centrifuged at 6000 g for 15 min, and the absorbance was measured at 410 nm (Wang et al., 2014).

MDA content: 0.5 g fresh leaves were ground into a homogenate in 2 ml of 10% trichloroacetic acid and centrifuged at 4000 g, 4°C for 10 min. Supernatant (2 ml) was mixed with 0.6% TBA. Afterward, the mixture was boiled in a water bath for 25 min, cooled to room temperature and then centrifuged again. The supernatant was taken and the absorbance was measured at 440 nm, 532 nm and 600 nm, respectively. The MDA concentration (μmol L^−1^) was calculated as 6.45 × (A_532_-A_600_)*−*0.56 × A_450_ (Talaat and Shawky, 2016).

Electrolyte leakage (EL): After cutting off the leaves and rinsing them with deionized water, the surface water was blotted out with filter paper. Then the leaves (1 g) were cut into small pieces (about 1 cm^2^), and soaked in deionized water (25°C, 2 ml) for 24 h. The initial conductivity (EC1) was determined using an electrical conductivity meter. Afterward, the samples were subjected to a boiling water bath for 10 min to completely kill the tissue and release all electrolytes, and cooled to 25°C. At last, the final conductivity (EC2) was determined. EL was calculated by the formula: EL = the initial conductivity (EC1)/the final conductivity (EC2) × 100 (Altaf et al., 2022b).

Antioxidant enzyme activity: Fresh leaf sample (0.5 g) was added into pre-cooled PBS (50 mM, pH 7.8, 5 ml) containing 1% PVP (polyvinylpyrrolidone) in a mortar and ground into a homogenate under ice bath. The homogenate was centrifuged at 10000 g, 4°C for 15 min and the supernatant was collected for the determination of antioxidant enzyme activity. SOD and POD activity was analyzed according to the nitrogen blue tetrazolium method and guaiacol, respectively. As CAT was able to decompose H_2_O_2_, CAT activity could be measured according to the determination of the decomposition rate of H_2_O_2_ (Sun et al., 2013). APX, MDHAR, DHAR, and GR enzyme activities were determined with literature methods (Ding et al., 2007; Guo et al., 2011).

AsA, DHA, GSH and GSSG content: AsA and DHA content were determined by Acid (AsA) Content Assay Kit (Beijing box Shenggong Technology Co., Ltd., Beijing, China). GSH and GSSG were determined by HPLC (Han et al., 2012; Mcgill and Jaeschke, 2015).

### Measurement of polyamine metabolism substances contents and enzymes activities

Endogenous polyamine content: 0.3 g fresh leaf sample was ground into a homogenate in pre-cooled perchloric acid (PCA, 4 ml, 5% V/V) and then kept at 4°C for 1 h. Then 1, 6 - hexanediamine was added to the homogenate as an internal standard and the mixture was centrifuged at 12000 g, 4°C for 30 min. The supernatant was used for the determination of free and soluble conjugated polyamine, while the precipitate was used for insoluble bound polyamine determination (Yuan et al., 2014; Li et al., 2018). The polyamine content was determined by high performance liquid chromatography (HPLC). After benzoylating, 20 μl of the sample was separated using a 5 × 4.6 × 250 mm C_18_ reversed-phase chromatographic column. The column temperature was maintained at 25°C, and the sample was eluted with 64% methanol at a flow rate of 0.8 ml min^−1^. The absorbance values were measured at 254 nm. The acidic soluble polyamine content was subtracted from the free polyamine content to calculate the soluble conjugated polyamine content (Verslues et al., 2006; Duan et al., 2008).

Polyamine synthase activity: 0.3 g fresh leaf sample was ground into a homogenate in potassium phosphate buffer (100 mm, pH 8.0) containing 0.1 mm phenylmethylsulfonyl fluoride, 1 mM pyridoxal phosphate (PLP), 5 mm EDTA, 25 mm ascorbic acid, and 0.1% polyvinylpyrrolidone. Then it was centrifuged at 12000 g, 4°C for 40 min. The supernatant was dialyzed against potassium phosphate buffer (3 ml, 100 mm, pH 8.0) containing 0.05 mm PLP, 0.1 mm DTT and 0.1 mm EDTA for 24 h at 4°C under dark conditions, which was used for enzyme assays. ADC was determined by benzoylation UV detection. ODC was detected by ELISA Kit for Ornithine (Shanghai Xinyu Biotechnology Co., Ltd., Shanghai, China), and SAMDC was determined by ELISA (Nanjing Camillo Bioengineering Co., Ltd., Nanjing, China; Jeoung et al., 2010).

Polyamine oxidase activity: 0.3 g fresh leaf sample was ground into a homogenate in K-phosphate buffer (100 mm, pH 6.5) containing 5 mm dithiothreitol, and the extracts were centrifuged at 16000 g, 4°C for 20 min. The supernatant was used for the determination of enzyme activity. Put and Spd were used as substrates to determine the DAO and PAO activities, respectively, according to research methods (Li et al., 2018).

### Data analysis

According to the analysis of variance, data were statistically analyzed following standard methods using Microsoft Excel 2010 and SPSS 12.0. Differences between treatments were determined by *a posteriori* Tukey’s test at a significance level of 0.05.

## Results

### Leaf photosynthetic pigment content and its ratio

Table 1 showed that Hemin can increase the *Chla*, *Chlb* and *Chl(a + b)* contents of the two varieties of maize leaves under normal moisture conditions. Compared with CK, the *Chla*, *Chlb*, *Chl(a + b)* content and *Chla*/*Chlb* ratio of leaves after Cd treatment for 4 days showed a significant decline, and the magnitude of the decline was related to maize genotype differences, with a greater decrease in Fenghe 6. Compared with Cd treatment, Hemin+Cd treatment significantly increased the photosynthetic pigment content of the two varieties of maize seedling leaves, and the increase in Fenghe 6 was greater than in Tiannong 9. Compared with Cd treatment, the *Chla*, *Chlb*, *Chl(a + b)* content and *Chla*/*Chlb* ratio of Tiannong 9 increased by 48.36, 21.3, 22.5 and 40.9%, respectively, after Hemin+Cd treatment; Fenghe 6 increased by 56.1, 39.4, 11.9 and 50.9%, respectively. This result showed that Hemin could promote the chlorophyll synthesis ability of leaves and slow down the decomposition and transformation process of chlorophyll under Cd stress, thereby enhancing the photosynthesis ability of maize leaves under Cd stress, and having better alleviation for Fenghe 6. Our studies showed that the *Car* content in maize leaves under Cd treatment decreased, while the *Car*/*Chl(a + b)* ratio increased significantly. Hemin+Cd treatment can slow down the decline of carotenoid content in leaves, and increase the ratio of *Car*/*Chl(a + b)* under Cd treatment. For example, compared with CK, the Car content of Tiannong 9 and Fenghe 6 treated with Hemin increased by 9.6 and 9.9%, and the ratio of *Car*/*Chl(a + b)* increased by 3.7 and 4.9%, respectively. Compared with Cd treatment, the *Car* content of Tiannong 9 and Fenghe 6 under Hemin+Cd treatment increased by 52.8 and 68.8%, respectively, and the ratio of *Car*/*Chl(a + b)* increased by 8.1 and 12.3%, respectively (Table 1).

**Table 1:** Effects of Hemin on different photosynthetic pigment contents in maize seedling leaves under Cd stress (at the 4th day).

Varieties	Treatments	*Chla*	*Chlb*	*Chla/chlb*	*Chl(a + b)*	*Car*	*Car/Chl(a + b)*
Tiannong9	CK	2.872 ± 0.078b	0.811 ± 0.043b	3.547 ± 0.102a	3.683 ± 0.105b	0.397 ± 0.016b	0.108 ± 0.005b
	Hemin	3.349 ± 0.064a	0.914 ± 0.045a	3.670 ± 0.106a	4.263 ± 0.106a	0.478 ± 0.036a	0.112 ± 0.006b
	Cd	1.253 ± 0.058d	0.474 ± 0.038d	2.646 ± 0.079c	1.728 ± 0.089d	0.214 ± 0.034d	0.124 ± 0.005a
	Hemin+Cd	1.859 ± 0.077c	0.575 ± 0.033c	3.241 ± 0.121b	2.434 ± 0.098c	0.327 ± 0.025c	0.134 ± 0.006a
Fenghe6	CK	2.677 ± 0.058b	0.795 ± 0.033b	3.372 ± 0.124a	3.472 ± 0.068b	0.374 ± 0.018b	0.103 ± 0.006
	Hemin	3.095 ± 0.095a	0.907 ± 0.028a	3.412 ± 0.112a	4.002 ± 0.121a	0.411 ± 0.021a	0.108 ± 0.003
	Cd	0.880 ± 0.087d	0.376 ± 0.036d	2.342 ± 0.068c	1.257 ± 0.068d	0.144 ± 0.016d	0.114 ± 0.003a
	Hemin+Cd	1.374 ± 0.124c	0.524 ± 0.034c	2.621 ± 0.104b	1.898 ± 0.107c	0.243 ± 0.015c	0.128 ± 0.004a

Means within a column followed by the different letters indicate a significant difference at *p* < 0.05.

### Lutein cycle components

As shown in Table 2, compared with CK, Hemin increased the content of V, A, Z and VAZ and the value of DEPS in leaves of the two varieties of maize. The contents of V, A, Z and VAZ and DEPS values in leaves of Tiannong 9 treated with Hemin increased by 7.96, 34.1, 28.2, 12.6 and 15.9%, respectively, compared with CK. And the contents of V, A, Z and VAZ and the value of DEPS in Fenghe 6 leaves treated with Hemin increased by 13.6, 39.1, 50.9, 18.8 and 22.7%, respectively. Cadmium stress significantly decreased the content of V and total V + A + Z in the leaf lutein cycle. For example, the V content and the total V + A + Z content of Tiannong 9 reach 25.45 and 46.62 mmol mol^−1^ chl, respectively. Nevertheless, the content of A and Z increased, and those of Tiannong 9 reached 7.71 and 13.455 mmol mol^−1^ chl, respectively. Tiannong 9 after Cd treatment had higher V, A, Z and VAZ content and DEPS than Fenghe 6. After Hemin+Cd treatment, the content of V, A, Z and VAZ in maize leaves and the value of the oxidation state DEPS of the lutein cycle increased. Compared with Cd treatment, the contents of V, A, Z, VAZ and DEPS in leaves of Tiannong 9 under Hemin+Cd treatment increased by 23.2, 73.5, 180.2, 76.8 and 36.5%, respectively, and the V, A, Z, VAZ content and DEPS of Fenghe 6 increased by 42.02, 75.1, 174.8, 78.8 and 30.91%, respectively. The results showed that exogenous Hemin improved the ratio of pigments in the lutein cycle and its physiological transformation process under Cd stress, and played an essential role in eliminating excitation energy and avoiding light damage in PSII (Table 2).

**Table 2:** Effects of Hemin on xanthophyll cycle components in maize seedling leaves under Cd stress (at the 4th day).

Varieties	Treatments	V (mmol.mol^−1^chl)	A (mmol.mol^−1^chl)	Z (mmol.mol^−1^chl)	V + A + Z (mmol.mol^−1^chl)	(A + Z)/(V + A + Z) (DEPS value)
Tiannong9	CK	45.813 ± 0.859a	4.496 ± 0.589c	7.247 ± 0.368c	57.556 ± 0.785b	0.204 ± 0.004c
	Hemin	49.459 ± 0.748a	6.029 ± 0.411c	9.292 ± 0.752c	64.780 ± 0.965b	0.237 ± 0.008c
	Cd	25.455 ± 0.695c	7.710 ± 0.124b	13.455 ± 0.634b	46.620 ± 0.785c	0.454 ± 0.010b
	Hemin+Cd	31.350 ± 0.574b	13.379 ± 0.242a	37.708 ± 0.469a	82.437 ± 0.855a	0.620 ± 0.011a
Fenghe6	CK	40.415 ± 0.745a	3.435 ± 0.256c	4.422 ± 0.552c	48.272 ± 0.874b	0.163 ± 0.003c
	Hemin	45.891 ± 0.663a	4.775 ± 0.272c	6.671 ± 0.341c	57.337 ± 0.955b	0.200 ± 0.007c
	Cd	21.470 ± 0.489c	5.843 ± 0.338b	8.444 ± 0.716b	35.757 ± 0.741c	0.400 ± 0.007b
	Hemin+Cd	30.491 ± 0.579b	10.232 ± 0.741a	23.210 ± 0.645a	63.933 ± 0.748a	0.523 ± 0.004a

Means within a column followed by the different letters indicate a significant difference at *p* < 0.05.

### Gas exchange parameters

As shown in Table 3, after Cd treatment for 4 days, the *P_n_* values of leaves showed a significant downward trend compared with CK, with a decrease of 45.1 and 58.6% in Tiannong 9 and Fenghe 6, respectively. There were genotype differences in the response of different varieties to Cd stress, with a higher decrease for Fenghe 6 than for Tiannong 9. Compared with Cd treatment, Hemin+Cd treatment significantly increased *P_n_*, with 50.04 and 82.16% increases in Tiannong 9 and Fenghe 6, respectively, and the increase in Fenghe 6 was more significant than that in Tiannong 9. Compared with CK, the *P_n_* parameter value of leaves increased after Hemin treatment, and the *P_n_* parameter value of different Cd stress types of maize had different responses to Hemin, which showed that the *P_n_* value of Tiannong 9 was higher than that of Fenghe 6. The changing trend of *g_s_* after Hemin+Cd treatment was consistent with the trend of *T*_r_, showing an increase in stomatal conductance and transpiration rate, which was consistent with that of *P_n_*. For example, Hemin+Cd treatment significantly increased the *g*_s_ and *T*_r_ of two varieties of maize seedlings compared with Cd treatment. Tiannong 9 increased by 26.33 and 32.41%, and Fenghe 6 increased by 41.24 and 42.16%, respectively, and Fenghe 6 was greater than that of Tiannong 9. Hemin+Cd treatment significantly increased the *C_i_* in the leaves of Tiannong 9 and significantly decreased the *C_i_* in the leaves of Fenghe 6 under Cd stress. Compared with Cd treatment, *C_i_* in leaves of Tiannong 9 increased by 23.22%, and *C_i_* in leaves of Fenghe 6 decreased by 14.26% treated with Hemin. The change trend of the stomatal restriction (*L*_s_) characteristics of the two varieties of leaves was opposite to that of *C_i_* under Cd treatment. Further data analysis showed that Hemin+Cd treatment significantly increased the leaf *L*_s_ of Fenghe 6, but decreased the leaf *L*_s_ of Tiannong 9 *L*_s_. In terms of water use efficiency (WUE), the WUE of Tiannong 9 under the same treatment level is higher than that of Fenghe 6. Compared with CK, the WUE of seedling leaves after Cd treatment was significantly increased, which was increased by 31.44 and 18.32% in Tiannong 9 and Fenghe 6, respectively. The results showed that Cd treatment inhibited photosynthesis of maize seedlings, while Hemin+Cd treatment improved the gas exchange parameter values of maize leaves under Cd stress. It specifically demonstrated that exogenous Hemin increased leaf *P_n_*, *g_s_*, *T_r_*, *L*_s_ and WUE, and reduce the *C_i_* of leaves, with differences among varieties, that is, the effect of improving the gas exchange parameters of the leaves of Fenghe 6 intolerant to Cd stress is more prominent (Table 3).

**Table 3:** Effects of Hemin on gas exchange parameters and chlorophyll fluorescence parameters in maize seedling leaves under Cd stress.

Varieties	Treatments	Gas exchange parameters						Chlorophyll fluorescence parameters					
		*P_n_* (μmol m^−2^ s^−1^)	*g_s_* (μmol m^−2^ s^−1^)	*T_r_* (mmol m^−2^ s^−1^)	WUE	*L_s_*	*C*_i_ (μmol mol^−1^)	*F_m_*	*F_v_/F_m_*	*ФPSII*	*ETR*	*qP*	*NPQ*
Tiannong9	CK	14.89 ± 0.37a	0.085 ± 0.002a	4.56 ± 0.20a	3.27 ± 0.21c	0.451 ± 0.025c	219.69 ± 9.32a	1.890 ± 0.05a	0.822 ± 0.020b	0.703 ± 0.029b	88.6 ± 3.75a	0.907 ± 0.04b	0.296 ± 0.012c
	Hemin	16.16 ± 0.41a	0.086 ± 0.002a	4.48 ± 0.27a	3.56 ± 0.20c	0.415 ± 0.024c	234.14 ± 9.80a	1.924 ± 0.04a	0.850 ± 0.010a	0.810 ± 0.027a	91.0 ± 3.30a	0.960 ± 0.01a	0.331 ± 0.034c
	Cd	7.83 ± 0.45c	0.057 ± 0.004c	1.77 ± 0.09c	4.43 ± 0.16b	0.632 ± 0.027a	147.08 ± 10.81c	1.010 ± 0.02c	0.628 ± 0.023d	0.360 ± 0.030d	40.9 ± 3.87c	0.601 ± 0.02d	0.471 ± 0.024b
	Hemin+Cd	12.00 ± 0.52b	0.073 ± 0.003b	2.40 ± 0.10b	4.99 ± 0.07a	0.536 ± 0.027b	185.63 ± 10.71b	1.150 ± 0.04b	0.750 ± 0.018c	0.514 ± 0.027c	65.2 ± 3.50b	0.763 ± 0.02c	0.682 ± 0.035a
Fenghe6	CK	13.72 ± 0.55a	0.080 ± 0.004a	4.72 ± 0.12a	2.91 ± 0.21c	0.470 ± 0.023a	211.94 ± 10.50c	1.680 ± 0.06a	0.812 ± 0.013a	0.617 ± 0.028b	78.5 ± 3.54a	0.808 ± 0.03b	0.298 ± 0.027c
	Hemin	15.48 ± 0.48a	0.086 ± 0.006a	4.76 ± 0.12a	3.27 ± 0.20b	0.427 ± 0.032a	229.24 ± 11.20c	1.650 ± 0.05a	0.840 ± 0.020a	0.732 ± 0.019a	84.0 ± 3.61a	0.922 ± 0.01a	0.336 ± 0.036c
	Cd	5.24 ± 0.54c	0.040 ± 0.001c	1.52 ± 0.13c	3.44 ± 0.13b	0.162 ± 0.030c	335.06 ± 10.30a	0.862 ± 0.06c	0.445 ± 0.024c	0.223 ± 0.025d	33.2 ± 2.88c	0.507 ± 0.02d	0.432 ± 0.041b
	Hemin+Cd	9.90 ± 0.50b	0.058 ± 0.009b	2.18 ± 0.07b	4.53 ± 0.22a	0.301 ± 0.024b	279.72 ± 9.59b	1.117 ± 0.06b	0.620 ± 0.025b	0.410 ± 0.019c	48.0 ± 3.30b	0.710 ± 0.02c	0.562 ± 0.038a

Means within a column followed by the different letters indicate a significant difference at *p* < 0.05.

### Chlorophyll fluorescence parameters

As shown in Table 3, compared with CK, Hemin increased *F_v_/F_m_* and *ФPSII* in two varieties of maize seedlings, and also increased *ETR* and *qP* in Fenghe 6. After Cd treatment for 4 days, *F_m_* and *F_v_/F_m_* values decreased, significantly lower than CK. And the values of *ФPSII* and *ETR* also showed a downward trend. The *qP* value decreased significantly, but the *NPQ* value increased significantly (*p* < 0.05). Cadmium stress inhibited the increase of the chlorophyll fluorescence parameters of maize leaves, which was extremely unfavorable to the light energy absorption and utilization of leaves. Compared with CK, *F_m_*, *F_v_/F_m_*, *ФPSII*, *ETR* and *qP* in Tiannong 9 after Cd treatment decreased by 46.7, 23.6, 48.8, 53.9 and 33.8%, respectively, and decreased by 48.7, 45.2, 63.8, 57.8 and 36.4%, respectively, in Fenghe 6. Compared with normal conditions, the *NPQ* of Tiannong 9 and Fenghe 6 under Cd treatment increased by 59.1 and 44.8%, respectively. After Hemin+Cd treatment, the *F_m_*, *F_v_/F_m_*, *ФPSII*, *ETR*, *qP* and *NPQ* values of the two varieties of maize seedlings increased. Compared with Cd treatment, Hemin+Cd treatment increased the chlorophyll fluorescence value of Tiannong 9 leaves. Taking Tiannong 9 as an example, *F_m_*, *F_v_/F_m_*, *ФPSII*, *ETR*, *qP* and *NPQ* increased by 85.1, 18.4, 42.9, 59.7, 27.1 and 44.8%, respectively (Table 3).

### RUBPCase and PEPCase activities

**Figure 1:**
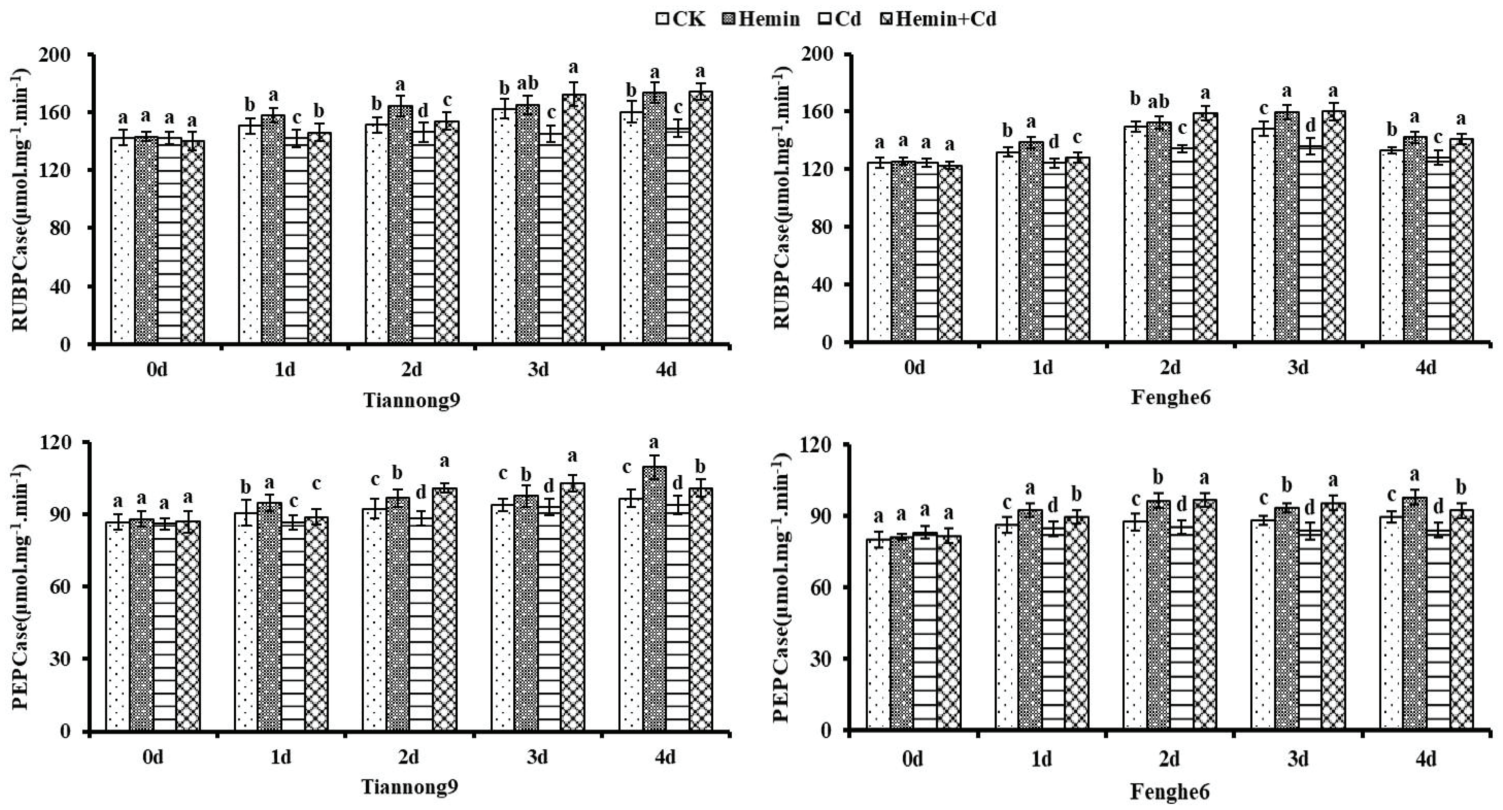
Effects of Hemin on activities of RUBPCase and PEPCase in maize seedling leaves under Cd stress. Data are expressed as mean ± standard deviation. Different letters within the same column indicate significant difference at 5% level.

As shown in Figure 1, the RUBPCase and PEPCase activities of leaves treated with Hemin increased compared with CK. Taking the 4th day as an example, the leaf enzyme activity of RUBPCase of Tiannong 9 and Fenghe 6 was 8.3 and 6.8% higher than that of CK, respectively, and the leaf enzyme activity of PEPCase of those was 12.9 and 9.2% higher than that of CK, respectively. After 1st to 4th day of Cd treatment, the RUBPCase and PEPCase activities of maize leaves decreased, and the photosynthetic capacity of the leaves decreased, with a higher decrease in photosynthetic enzyme activity of the leaves in Fenghe 6. Compared with CK, the RUBPCase activity of Tiannong 9 decreased by 5.71, 3.3, 10.5 and 7.05%, respectively, and the PEPCase activity decreased by 4.3, 4.11, 0.95 and 2.59%, respectively, at 1st, 2nd, 3rd and 4th day after Cd treatment. Compared with CK, the RUBPCase activity of Fenghe 6 at the 1st, 2nd, 3rd and 4th day after Cd treatment decreased by 5.87, 10.2, 7.96 and 3.87%, respectively, and PEPCase enzyme activity decreased by 1.86, 2.62, 5.1 and 6.13%, respectively. After Hemin+Cd treatment, the activities of RUBPCase and PEPCase in the two varieties’ leaves increased, significantly higher than that of Cd treatment. From the 1st to the 4th day of the experimental treatment, the photosynthetic enzyme activity value after Hemin+Cd treatment was significantly higher than that of CK and Cd treatment, indicating that Hemin can help maintain a robust carbon assimilation process of leaves under adversity, which was especially important for the normal function of the blade. Taking the 4th day as an example, the RUBPCase enzyme activity in leaves of Tiannong 9 was increased by 18.91% and the PEPCase enzyme activity was increased by 7.32% after Hemin+Cd treatment compared with Cd treatment, while the RUBPCase enzyme activity in leaves of Fenghe 6 was increased by 10.3% and the PEPCase enzyme activity was increased by 9.74%. From the perspective of physiology and biochemistry, Cd stress in this study reduced the activities of RUBPCase and PEPCase in the leaves, which also directly led to the decrease of the leaf gas exchange parameter *P_n_*. It showed that Cd stress not only destroyed the chloroplast membrane structure, but also inhibited the key photosynthetic enzyme activities, which resulted in a decrease in the photosynthetic rate of leaves and weakening of material accumulation and transformation ability (Figure 1).

### TBARS contents

Compared with CK, on the 1st, 2nd, 3rd and 4th day after Cd treatment, the TBARS content of Tiannong 9 leaves was 78.03, 147.7, 174.6 and 218.04% higher, respectively, and the TBARS content of the leaves of Fenghe 6 was 78.4, 155.7, 187.1 and 223.5% higher, respectively. There are genotypic differences in TBARS content among different varieties. When Tiannong 9 with strong Cd tolerance was exposed to Cd stress, more TBARS accumulated in its seedlings than Fenghe 6 with weak Cd tolerance. Under Cd treatment, the TBARS content in Tiannong 9 was 24.7, 6.4, 3.37 and 6.28% higher than that of Fenghe 6 at 1st, 2nd, 3rd and 4th day after treatment, respectively. Compared with Cd treatment, on the 1st, 2nd, 3rd and 4th day after Hemin+Cd treatment, the TBARS content in leaves of Tiannong 9 decreased by 7.66, 16.6, 9.78 and 9.93%, respectively, and decreased by 17.04, 176.3, 12.36 and 11.56%, respectively, in leaves of Fenghe 6. The results showed that Hemin effectively reduced the content of membrane lipid peroxidation TBARS in leaf tissues and the degree of membrane lipid peroxidation, and ensured the integrity of cell membranes under Cd stress (Table 4).

**Table 4:** Effects of Hemin on TBARS accumulations in maize seedling under Cd stress.

Varieties	Treatments	TBARS contents (μmol.g^−1^FW)				
		0d	1d	2d	3d	4d
Tiannong9	CK	1.33 ± 0.02a	1.32 ± 0.01c	1.34 ± 0.02c	1.34 ± 0.02c	1.33 ± 0.01c
	Hemin	1.32 ± 0.01a	1.04 ± 0.01c	1.03 ± 0.01c	1.05 ± 0.02c	1.04 ± 0.01c
	Cd	1.33 ± 0.01a	2.35 ± 0.02b	3.32 ± 0.02b	3.68 ± 0.01b	4.23 ± 0.01b
	Hemin+Cd	1.32 ± 0.01a	2.17 ± 0.01a	2.77 ± 0.02a	3.32 ± 0.01a	3.81 ± 0.02a
Fenghe6	CK	1.22 ± 0.02a	1.25 ± 0.02c	1.22 ± 0.02c	1.24 ± 0.01c	1.23 ± 0.01c
	Hemin	1.21 ± 0.01a	1.11 ± 0.01c	1.13 ± 0.01c	1.12 ± 0.02c	1.15 ± 0.01c
	Cd	1.20 ± 0.01a	2.23 ± 0.02b	3.12 ± 0.02b	3.56 ± 0.01b	3.98 ± 0.02b
	Hemin+Cd	1.22 ± 0.02a	1.85 ± 0.02a	2.57 ± 0.02a	3.12 ± 0.01a	3.52 ± 0.01a

Means within a column followed by the different letters indicate a significant difference at *p* < 0.05.

### O_2_^−^· production rate and H_2_O_2_ content

**Figure 2:**
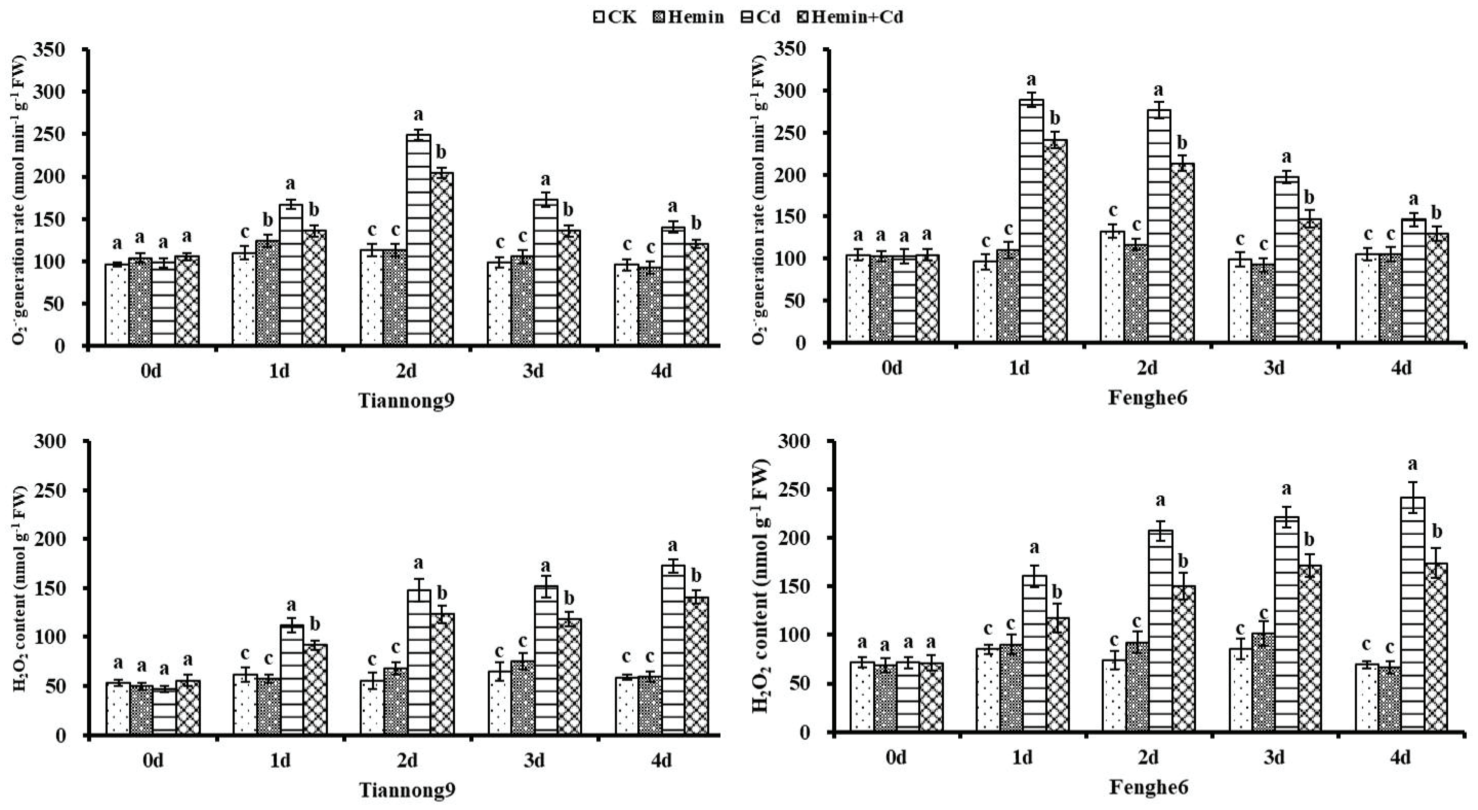
Effects of Hemin on leaf O_2_^−^·generation rate and H_2_O_2_ content in maize seedling leaves under Cd stress (at the 4th day). Data are expressed as mean ± standard deviation. Different letters within the same column indicate significant difference at 5% level.

As shown in Figure 2, the O_2_^−^· production rate and H_2_O_2_ content of Tiannong 9 increased by 33.2 and 142.5%, respectively, and those of Fenghe 6 increased by 41.6 and 221.4%, respectively, on the 4th day after Cd treatment compared with CK. Exogenous Hemin can significantly reduce the content of O_2_^−^· and H_2_O_2_ in leaves under Cd stress. Compared with Cd, the O_2_^−^· production rate in the leaves of Tiannong 9 and Fenghe 6 treated with Hemin+Cd decreased by 12.36 and 14.28%, respectively, while the H_2_O_2_ content in the two varieties decreased by 16.15 and 21.06%, respectively. This result indicated that Hemin could alleviate the production of reactive oxygen species in the leaves of maize seedlings induced by Cd stress, reduce O_2_^−^· production rate and H_2_O_2_ accumulation, thereby maintaining the integrity of cell membranes, and the enhancement effect was more significant for Fenghe 6, which was beneficial to the normal physiological functions of the leaves under adversity (Figure 2).

### EL, MDA and activities of SOD, POD, CAT

**Figure 3:**
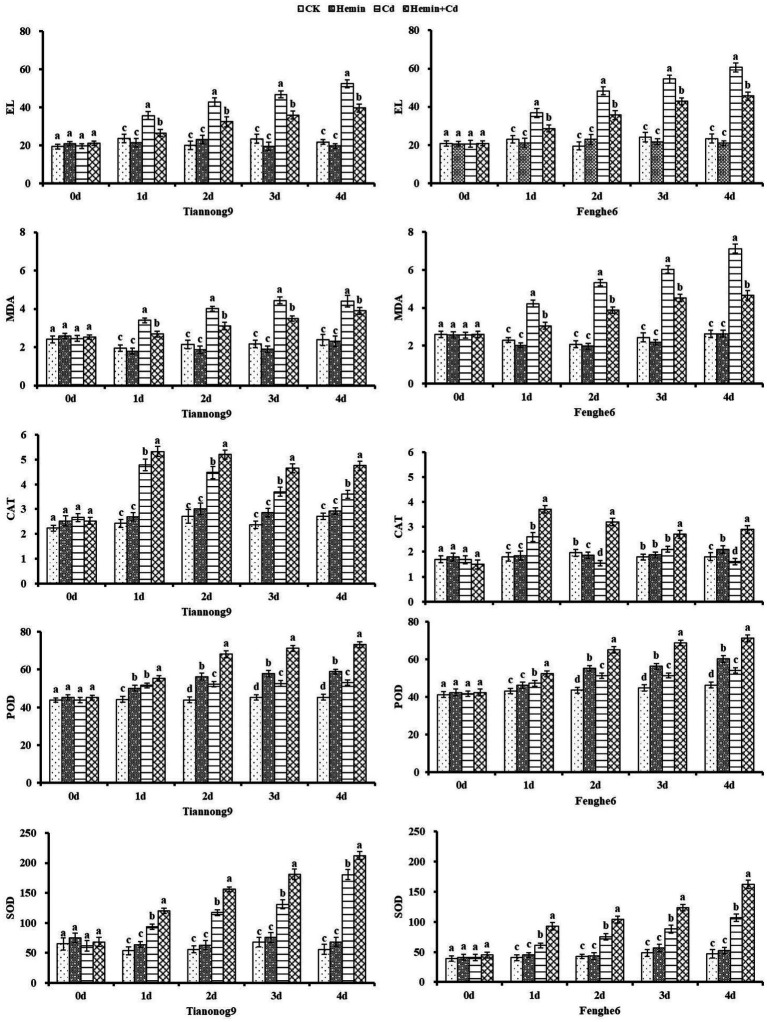
Effect of Hemin on EL, MDA contents, the activities of CAT, POD and SOD in maize seedling under Cd stress. Data are expressed as mean ± standard deviation. Different letters within the same column indicate significant difference at 5% level.

As shown in Figure 3 that Cd stress caused the leaf EL to show an increasing trend, which was manifested as a slow rise in the early stage and a sharp rise in the later stage, showing an increasingly severe trend as the stress level advanced. Compared with CK, on the 1st, 2nd, 3rd and 4th day after Cd treatment, the leaf EL content of Tiannong 9 was 51, 113.7, 100.4 and 140.9% higher, respectively, and the leaf EL content of Fenghe 6 was 60.1, 148.2, 127.4 and 160.1% higher, respectively. The EL was reduced by 24.5% for Tiannong 9 and 24.6% for Fenghe 6 at the 4th day after Hemin+Cd treatment compared with Cd treatment. This indicated that Hemin reduced the EL caused by Cd stress, and the low EL rate implied that the leaf tissue structure was intact and the leaf cell membrane was protected. The leaf MDA content of maize seedlings of both varieties continued to increase with increasing duration of Cd stress. Compared with CK, the leaf MDA content increased by 84.8% for Tiannong 9 at the 4th day of Cd treatment, while it increased by 171.4% for Fenghe 6. The MDA content in Tiannong 9 and Fenghe 6 leaves decreased by 11.36 and 34.45% after Hemin+Cd treatment compared with Cd stress. It showed that Hemin can effectively inhibit the damage of membrane lipid peroxidation to cell membrane under Cd stress, increase the level of MDA content and reduce the degree of EL, which protected the integrity of cell membrane.

From 1st to the 4th day, the SOD and POD activities of maize seedlings showed a gradual increasing trend, while CAT activity showed an inverted “V” single-peak trend of increasing first but then decreasing, and reached a high value on the 1st day after treatment. And the leaf SOD, POD and CAT enzyme activities had genotype differences under Cd stress. Compared with CK, on the 1st, 2nd, 3rd and 4th day after Cd treatment, the leaf SOD content for Tiannong 9 was 74.8, 109.4, 93.01 and 223.4% higher, respectively, and for Fenghe 6 was 50.6, 77.6, 82.1 and 126.5% higher, respectively. On the 1st, 2nd, 3rd and 4th days after Cd treatment compared with CK, the leaf POD content for Tiannong 9 was 16.7, 19.1, 16.6 and 17.2% higher, respectively, and for Fenghe 6 was 9.25, 17.7, 14.5 and 16.8% higher, respectively. The antioxidant enzyme activity was enhanced after Hemin treatment under Cd stress. Compared with Cd treatment, SOD enzyme activity of Tiannong 9 and Fenghe 6 leaves significantly increased by 33.3 and 37.8%,17.5 and 51.4%, respectively, and POD enzyme activity increased by 30.4 and 26.9%, 35.4 and 31.6%, respectively, and CAT enzyme activity increased by 16.4 and 107.8%, 16.4 and 32.1 and 81.3%, respectively. The results showed that the activities of antioxidant enzymes SOD, POD and CAT in leaves increased after exogenous Hemin treatment, and the ability of leaves to remove the large amount of O_2_^−^· and H_2_O_2_ produced under Cd stress increased, thus effectively alleviating leaf oxidation damage caused by Cd stress (Figure 3).

### AsA-GSH cycle enzyme activities and non-enzymatic antioxidants

**Figure 4:**
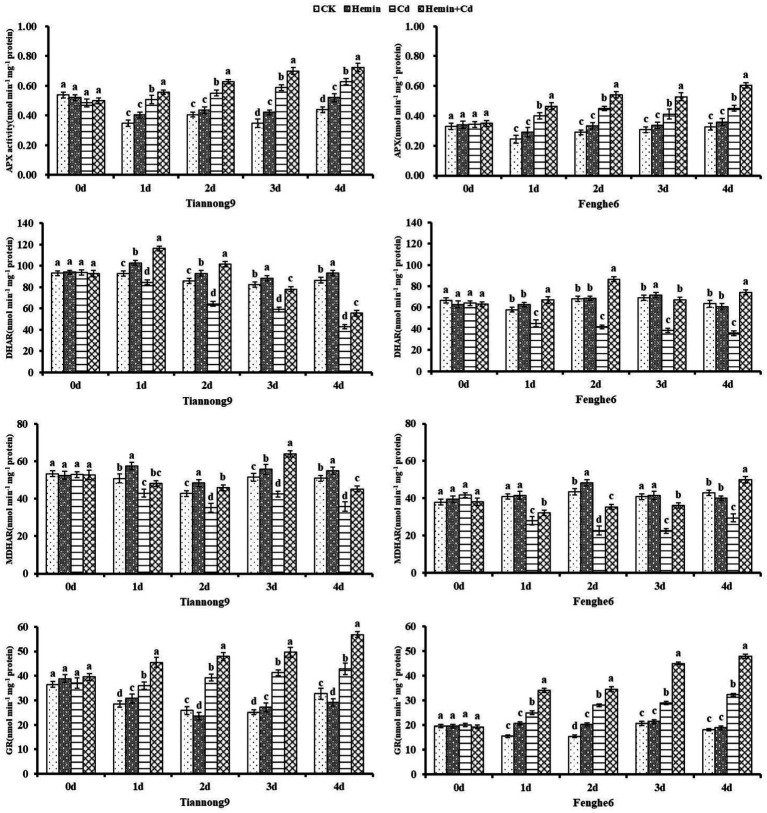
Effect of Hemin on AsA-GSH cycle key enzyme activity (APX, DHAR, MDHAR, GR) in maize seedling leaves under Cd stress. Data are expressed as mean ± standard deviation. Different letters within the same column indicate significant difference at 5% level.

As shown in Figure 4, APX and GR enzyme activities of maize leaves increased after Cd treatment, and DHAR and MDHAR enzyme activities decreased compared with CK. On the 1st, 2nd, 3rd and 4th day after Cd treatment compared with CK, the leaf APX activity for Tiannong 9 was 45.78, 35.63, 68.96 and 43.05% higher, respectively, and for Fenghe 6 was 63.77, 54.66, 34.04, and 37.8% higher, respectively. On the 1st, 2nd, 3rd and 4th day after Cd treatment compared with CK, the leaf GR enzyme activities for Tiannong 9 were 26.42, 51.76, 63.84 and 30.83% higher, respectively, and for Fenghe 6 was 61.31, 82.31, 39.79 and 77.18% higher, respectively. Hemin+Cd treatment increased the activity of the four cycles enzymes APX, MDHAR, DHAR and GR compared with Cd treatment. For example, compared with Cd treatment, on the 1st, 2nd, 3rd, and 4th day after Hemin+Cd treatment, MDHAR enzyme activity of leaves for Tiannong 9 increased by 37.42, 57.69, 32.44 and 29.5%, respectively, and for Fenghe 6 increased by 11.97, 30.37, 50.47 and 26.41%, respectively. This indicated that exogenous Hemin helped to maintain the AsA-GSH cycle in maize seedlings at a high operating efficiency under Cd stress, thereby improving the ability of maize seedlings to resist Cd stress.

**Figure 5:**
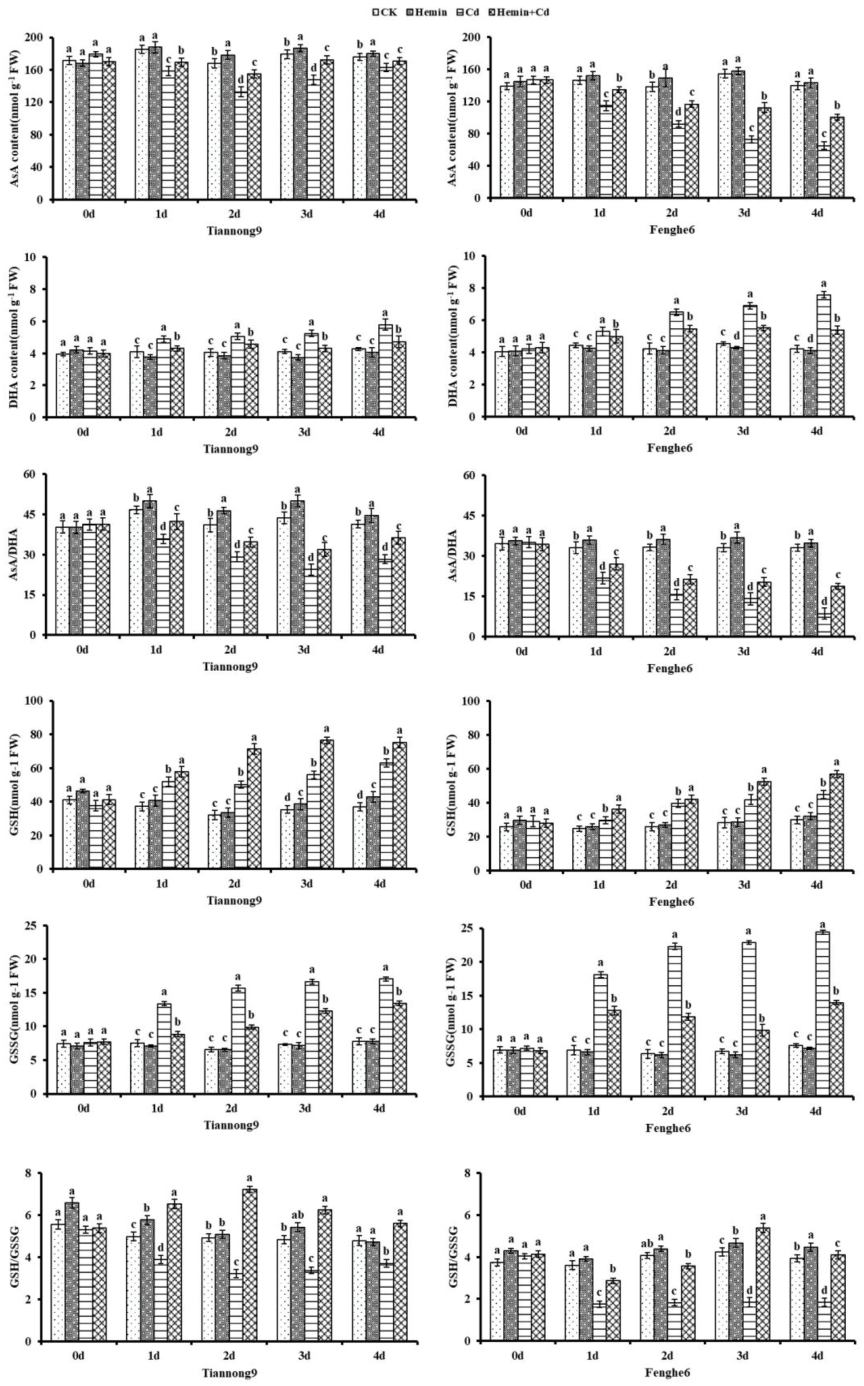
Effect of Hemin on the contents of AsA, DHA, GSH and GSSG and the ratio of AsA/DHA and GSH/GSSG in maize seedling leaves under Cd stress. Data are expressed as mean ± standard deviation. Different letters within the same column indicate significant difference at 5% level.

As shown in Figure 5, the AsA content in the leaves of Tiannong 9 showed a trend of first decreasing and then increasing from the 1st to the 4th day after Cd stress, and the AsA/DHA ratio presented similar characteristics to the change of AsA content. However, the AsA content and AsA/DHA ratio in Fenghe 6 leaves showed a downward trend, which may be caused by the genotypes of maize varieties with different Cd stress tolerance types. Compared with Cd treatment, Hemin+Cd treatment significantly increased the AsA content and AsA/DHA ratio in the leaves of the two varieties of maize seedlings. Taking the 4th day of Cd treatment as an example, compared with Cd treatment, the AsA content and the AsA/DHA ratio for Fenghe 6 treated with Hemin+Cd increased by 50.16 and 112.1%, respectively, and for Tiannong 9 increased by 4.7 and 28.3%, respectively. With the advancing of Cd stress time, the DHA content in the leaves increased, and the increase in Fenghe 6 was greater than that in Tiannong 9. Furthermore, Cd stress leads to a significant decrease in the ratio of AsA/DHA. The results showed that Hemin was beneficial to maintaining the stability of the cell’s internal environment and achieving the ability to eliminate ROS to resist stress conditions.

As the experimental treatment progressed from the 1st to the 4th day, the contents of GSH and GSSG in the two varieties of maize seedlings under the Cd stress treatment showed a gradually increasing trend, and reached the maximum after the 4th day of stress. Hemin+Cd treatment increased the GSH content of leaves and decreased the GSSG content compared with Cd treatment. For example, on the 1st, 2nd, 3rd, and 4th day after Hemin+Cd treatment compared with Cd treatment, GSH content of leaves for Tiannong 9 increased by 10.9, 41.7, 36.5 and 19.3%, respectively, and for Fenghe 6 increased by 22.4, 6.14, 25.5 and 27.3%, respectively. Hemin+Cd treatment increased the GSH content in leaves and slowed the increase of GSSG content simultaneously compared with Cd treatment, with differences among different varieties. The GSH/GSSG ratio of leaves after Cd treatment decreased significantly compared with CK, especially in Fenghe 6 (Figure 5).

### Endogenous polyamine content

**Figure 6:**
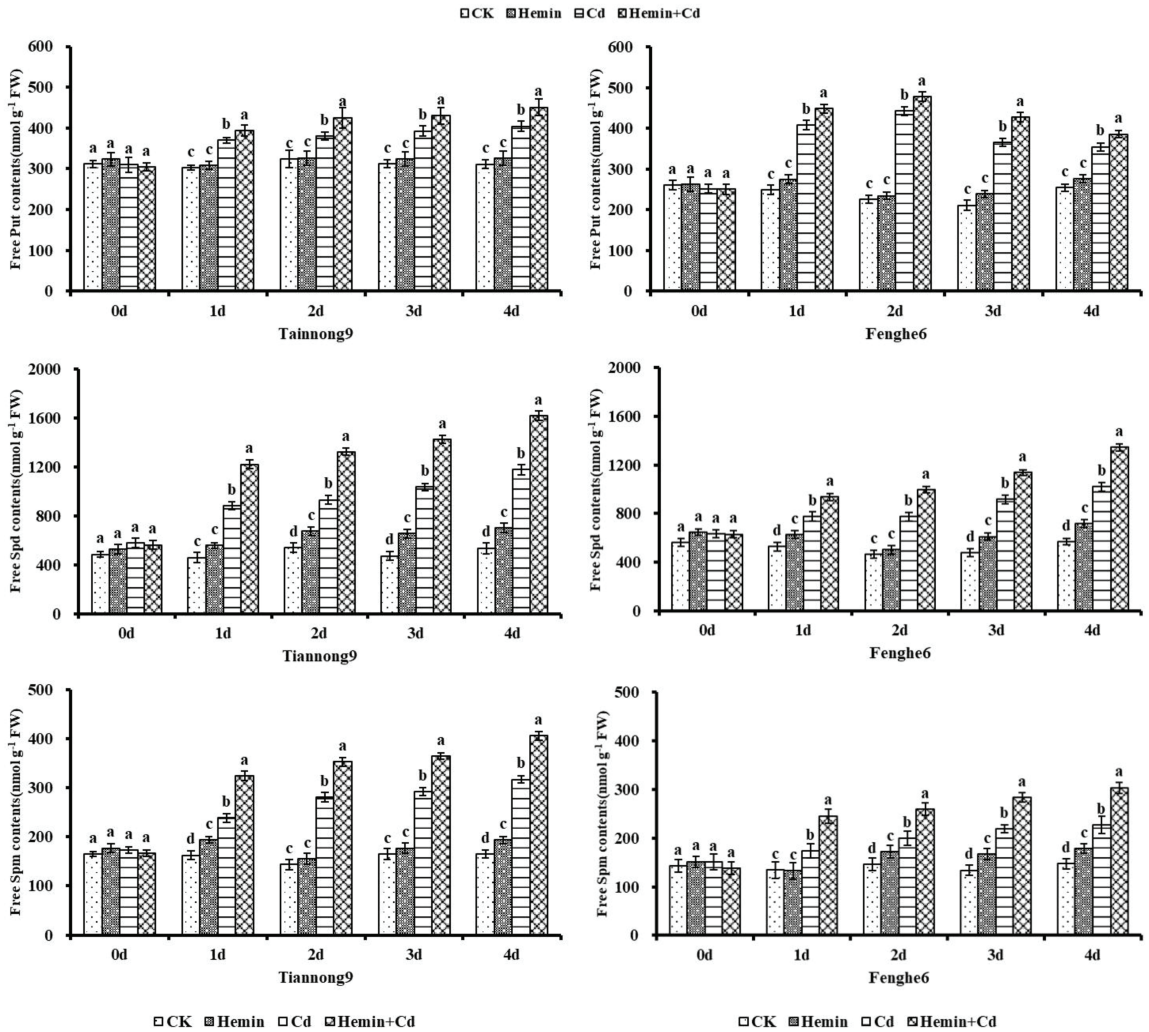
Effects of Hemin on free polyamine contents in maize seedling leaves under Cd stress. Data are expressed as mean ± standard deviation. Different letters within the same column indicate significant difference at 5% level.

As shown in Figure 6, the Put content increased after Cd and Hemin+Cd treatment, which was significantly higher than CK and Hemin treatment. For example, on the 4th day of experiment treatment, the Put content in leaves of Tiannong 9 and Fenghe 6 treated with Hemin+Cd was 450.56 and 385.67 nmol g^−1^ FW, respectively, which were 11.4 and 8.74% higher than those treated with Cd. The Spd content in the leaves of Tiannong 9 and Fenghe 6 treated with Hemin+Cd were 1619.75 and 1345.01 nmol g^−1^ FW, respectively, which were 37.5 and 31.9% higher than that of Cd treatment.

**Figure 7:**
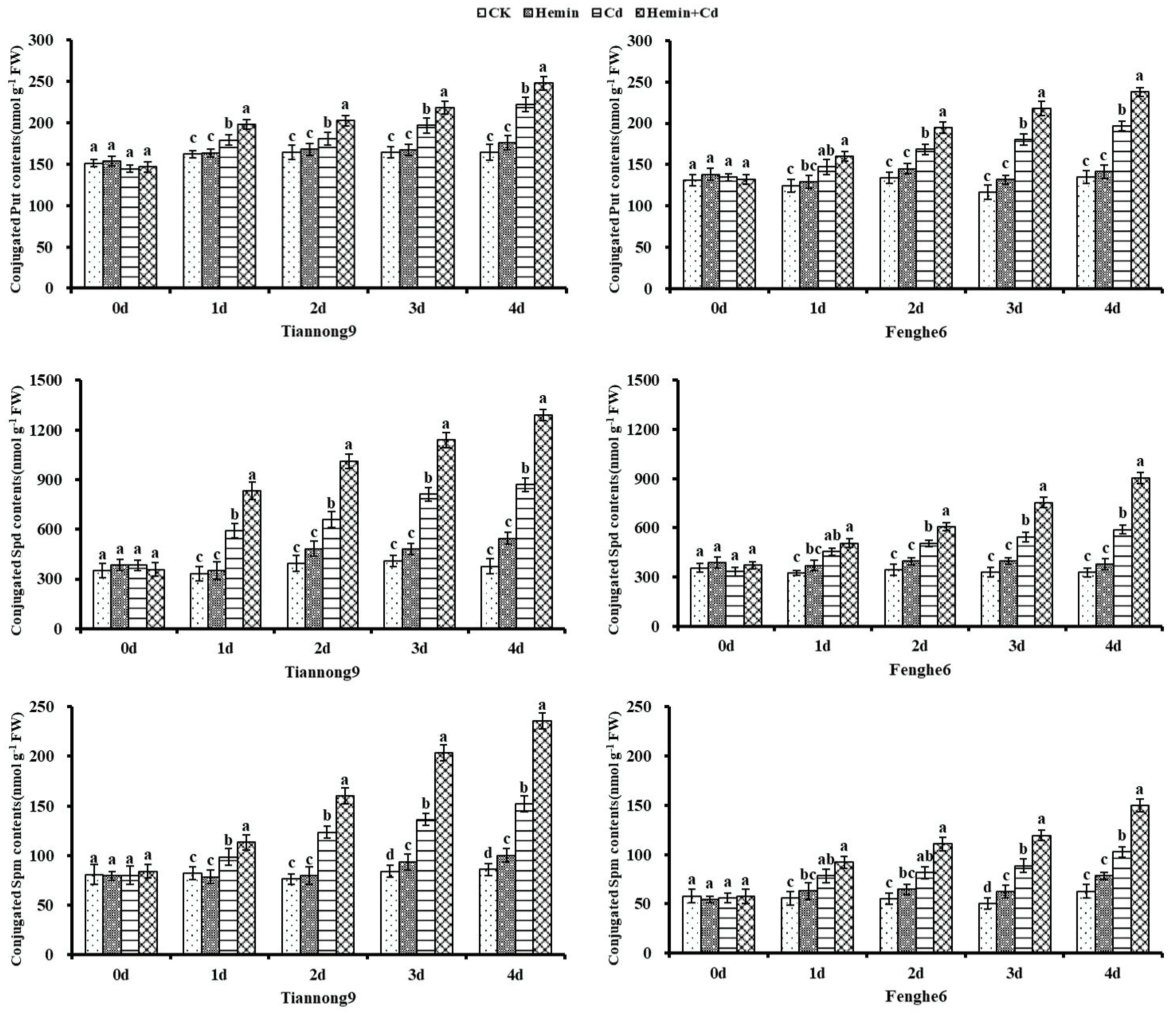
Effects of Hemin on conjugated polyamine contents in seedling leaves under Cd stress. Data are expressed as mean ± standard deviation. Different letters within the same column indicate significant difference at 5% level.

As shown in Figure 7, the conjugated polyamine content of the two maize seedlings from the 1st to the 4th day after Cd treatment showed a gradually increasing trend, and reached the maximum at the 4th day compared with CK. Taking the 4th day as an example, compared with Cd treatment, the conjugated Put content of Tiannong 9 and Fenghe 6 under Hemin+Cd treatment reached 248.11 and 237.89 nmol g^−1^ FW, respectively, which increased by 11.73 and 21.08%, respectively. Compared with CK, Hemin increased the content of conjugated polyamines to a certain extent, but the difference with CK was not significant, and its polyamines content was lower than that of Cd and Hemin+Cd treatment. This showed that exogenous Hemin further increased the conjugated polyamines content of the two varieties of maize seedlings under Cd stress.

**Figure 8:**
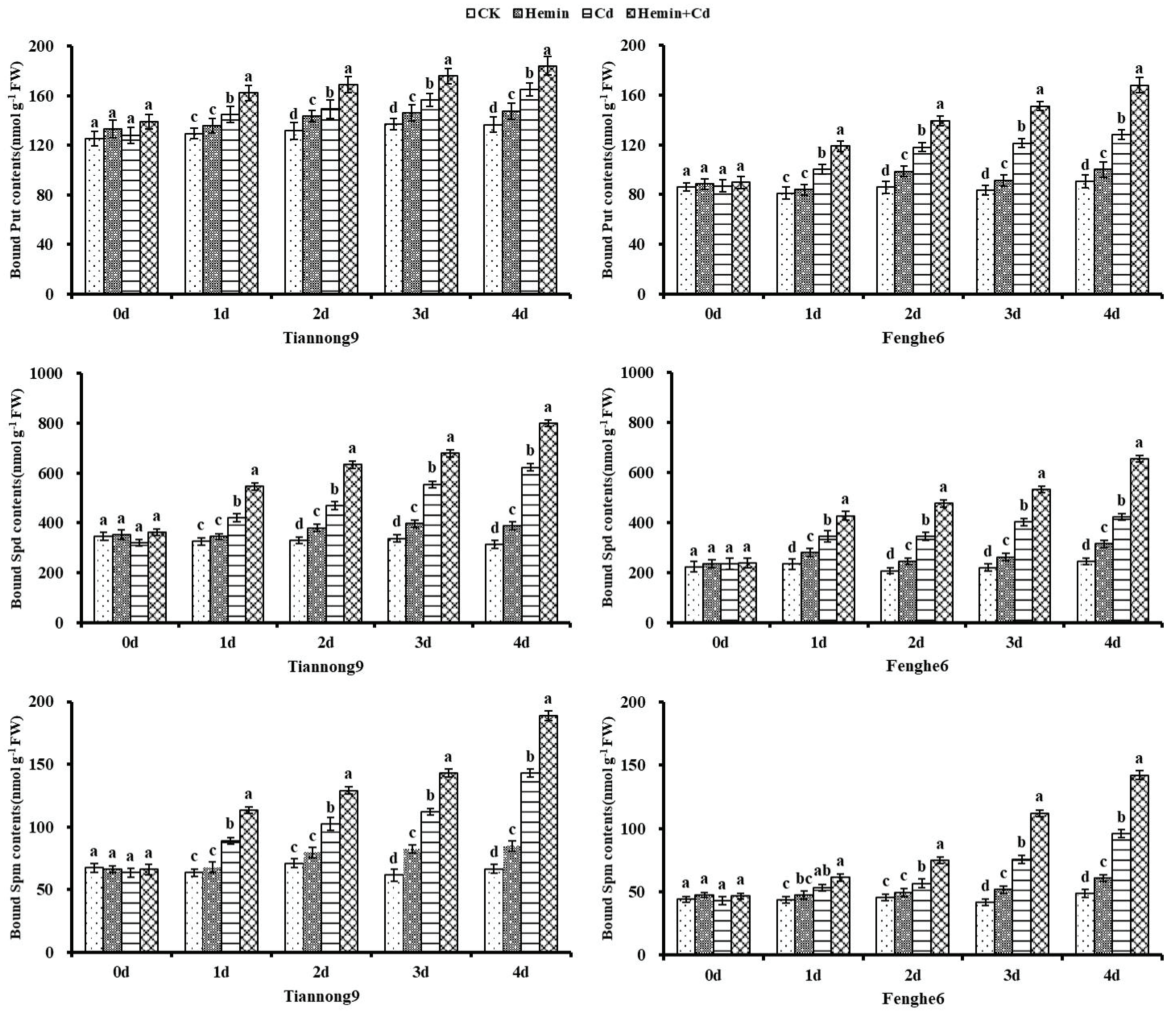
Effects of Hemin on bound polyamine contents in maize seedling leaves under Cd stress. Data are expressed as mean ± standard deviation. Different letters within the same column indicate significant difference at 5% level.

As shown in Figure 8, the content of bound polyamines from the 1st to the 4th day after the Cd treatment showed a gradual increase, and reached the maximum at the 4th day compared with CK. For example, on the 1st, 2nd, 3rd and 4th day after Cd treatment compared with CK, the leaf bound Put content for Tiannong 9 increased by 10.55, 10.04, 19.65 and 34.9%, respectively, and for Fenghe6 increased by 18.11, 25.73, 54.61 and 45.79%, respectively. Taking 4th day as an example, compared with Cd treatment, the bound Put content of Tiannong 9 and Fenghe6 under Hemin+Cd treatment reached 248.11 and 237.89 nmol g^−1^ FW, increasing by 11.73 and 21.08%, respectively. The bound Spd content of Tiannong 9 and Fenghe 6 reached 1290.81 and 904.04 nmol g^−1^ FW, increasing by 40.39 and 53.34%, respectively. The bound Spm content of Tiannong 9 and Fenghe 6 reached 23.5.78 and 149.97 nmol g^−1^ FW, increasing by 55.19 and 45.89%, respectively. Compared with CK, Hemin could increase the bound polyamines content to a certain extent, but the difference with CK was not significant, and its polyamines content was lower than Cd and Hemin+Cd treatment. This showed that exogenous Hemin further increased the bound polyamines content of the two varieties of maize seedlings under Cd stress.

### Activities of polyamine synthase and polyamine oxidase

**Figure 9:**
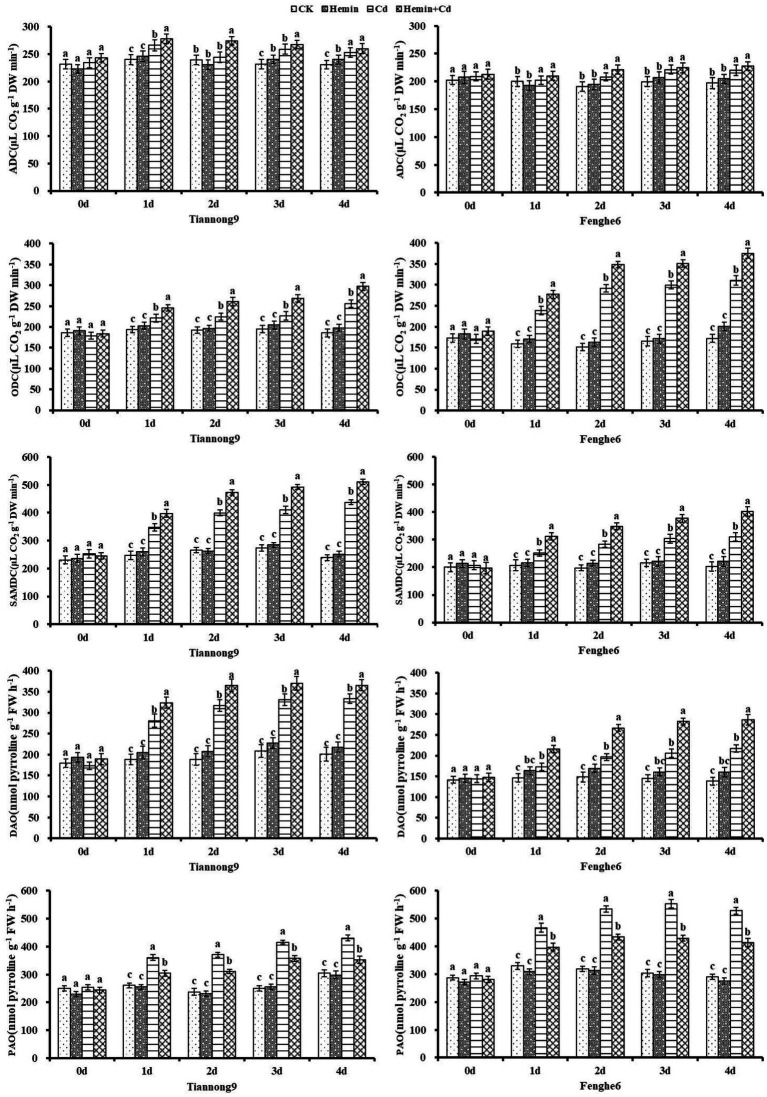
Effects of Hemin on the activities of ADC, ODC, SAMDC, DAO and PAO in maize seedling leaves under Cd stress (at the 4th day). Data are expressed as mean ± standard deviation. Different letters within the same column indicate significant difference at 5% level.

As shown in Figure 9, the ADC activity of maize leaves was significantly increased after Cd treatment compared with CK. For example, compared with CK, on the 1st, 2nd, 3rd and 4th day after Cd treatment, the ADC enzyme activity of leaves for Tiannong 9 increased by 10.88, 2.18, 11.68 and 9.5%, respectively, and for Fenghe6 increased by 1.33, 9.28, 11.09 and 11.86%, respectively. And under Cd stress, Hemin increased the ADC activity of the two maize varieties, but not significantly. For example, leaf ADC enzyme activity under Hemin+Cd treatment reached 259.83 and 227.72 nmol g^−1^ FW for Tiannong 9 and Fenghe 6, respectively, compared to Cd treatment, and increased by 2.8 and 3.34%, respectively, which were not significant. Cadmium stress significantly increased the activities of ODC and SAMDC in the leaves of the two varieties of maize seedlings, and Hemin enhanced the increase in ODC activity induced by Cd stress. The extent of the increase in ODC activity. Taking the 4th day as an example, compared with Cd treatment, the ODC activity in the leaves of Tiannong 9 and Fenghe6 under Hemin+Cd treatment reached 297.2 and 375.5 nmol g^−1^ FW, with an increase of 15.9 and 21.06%, respectively.

Cadmium stress significantly increased the activities of two amine oxidases of DAO and PAO, Hemin treatment increased the DAO activity but decreased PAO activity in plants under Cd stress, and this effect was more obvious in Fenghe 6. The DAO activity in Fenghe 6 leaves treated with Hemin for 4 days decreased by 31.69%compared with Cd stress, while the PAO activity increased by 22.77%. Based on the previous analysis of polyamines data, we suggest that the different levels and forms of PA accumulation in the two maize varieties should be at least partly attributed to the different responses of polyamines biosynthetic enzymes and catabolic enzymes to Cd stress. It also indicates that maize seedlings are tolerant to Cd stress, and the Cd stress tolerance of maize seedlings is related to the ability of seedlings to maintain high ADC, SAMDC, ODC and DAO enzyme activities and low PAO enzyme activity in response to Cd stress (Figure 9).

## Discussion

*Chla* and *Chlb* play a vital role in the absorption of light. In the excited state, *Chla* can convert light energy into electrical energy, and *Chlb* determines the wavelength of absorbable light (Wang et al., 2014). *Chla*/*Chlb* has an essential influence on the structural stability of LHCII. Carotenoids (*Car*) are also called auxiliary pigments, reducing the damage of solid light to photosynthetic mechanisms (Chorom et al., 2013). Compared with Cd treatment, Hemin+Cd treatment significantly increased the photosynthetic pigment content of the two varieties of maize seedling leaves, and the increase in Fenghe 6 was more significant than that of Tiannong 9. Our research indicated that Hemin can significantly alleviate the degradation or synthesis of chlorophyll in the leaves of maize seedlings caused by Cd stress, thereby enhancing the photosynthesis ability of maize leaves under Cd stress, with a better alleviating effect on Fenghe 6. Our study showed that the Car content in maize leaves under Cd treatment decreased, while the *Car*/*Chl(a + b)* ratio increased significantly. Hemin+Cd treatment slowed down the decline of carotenoid content in leaves and increased the ratio of *Car*/*Chl(a + b)* under Cd treatment. The results showed that exogenous Hemin significantly increased the chlorophyll, carotenoid content, and *Chla*/*Chlb* ratio in maize leaves, attributing to Hemin slowing down the decomposition of chlorophyll by regulating the enzyme activity related to chlorophyll biosynthesis and decomposition.

The lutein cycle plays a vital role in photoprotection (Baryla et al., 2001). Zeaxanthin Z can directly dissipate the excessive excitation energy absorbed by chlorophyll in the form of heat. In our study, Cd stress significantly reduced V and total lutein content (VAZ), increased A, Z content, and the ratio of (A + Z)/(V + Z + A). Exogenous Hemin increased the content of V, A, Z and VAZ, and increased the ratio of (A + Z)/(V + Z + A) in maize under Cd stress, thereby reducing the light damage caused by excessive excitation energy to photosynthetic organs (Maiti et al., 2012). Cadmium reduces the transcription process of photosynthesis-related genes (Souza et al., 2011). Most Cd poisoning can inhibit the photosynthesis of plants (Yuan et al., 2014). Photosynthesis is an important physiological process for maintaining plant growth and provides an interactive connection between the internal metabolism of plants and the external environment, which can reflect the changes of plants under environmental stress (Sun et al., 2013). In our study, Cd stress decreased *g*_*s*_ and *T_r_*, and stomata and/or non-stomata factors may cause the decrease of *P_n_*. In Fenghe 6, Cd stress decreased *g_s_* and *L_s_*, and increased *C_i_*, indicating that non-stomata restriction was the main reason for the decrease of *P_n_* in Fenghe 6 under Cd stress. In Tiannong 9, *C_i_* decreased while *L*_s_ increased, indicating that Cd stress inhibited the photosynthesis of Tiannong 9. Exogenous Hemin alleviated the decrease of *P_n_*, *g_s_, T_r_* and WUE, and inhibited the increase of *C*_i_ in Fenghe 6 leaves in maize leaves under Cd stress, which effectively alleviated the stomatal and non-stomata restriction.

When the light energy absorbed by LHCII is higher than the energy consumed by PSII, the over-excited energy will damage the photosynthetic organs of the plant, resulting in photoinhibition and photo injury, with PSII being most affected by photoinhibition (Li et al., 2018). Chlorophyll fluorescence can reflect the absorption, transmission, and conversion of light energy by leaves, which plays an essential role in the study of photosynthesis and stress physiology (Chen et al., 2018). Plants absorb light energy for photosynthesis, heat dissipation and fluorescence emission. *F_m_* is the maximum fluorescence, reflecting the electron transfer through PSII. *F_v_/F_m_* reflects the maximum photochemical quantum efficiency of PSII. In our study, Cd stress reduced the *F_m_* and *F_v_/F_m_* of maize seedlings and seriously affected the activity of the PSII reaction center. At the same time, the reduction of *ETR* produced excessive excitation energy, which aggravated the photoinhibition under Cd treatment (Xie et al., 2014). After 4 days of Cd stress, the *F_m_*, *F_v_/F*_*m*_, and *ETR* in Tiannong 9 were higher than Fenghe 6, indicating that the PSII reaction center in Tiannong 9 leaves could maintain high activity and keep open, thereby maintaining normal photochemical reactions. *qP* represents the energy used for photochemical electron transfer. In our study, the *qP* and *ΦPSII* values of Fenghe 6 seedlings were lower than those of Tiannong 9, and the PSII quantum yield was lower, indicating that Fenghe 6 could not effectively use the absorbed light energy and was easily affected by photoinhibition, and the PSII performance was better in Tiannong 9 under Cd stress. Exogenous Hemin significantly slowed down the reduction of *F_m_*, *F_v_/F_m_*, *ETR*, *qP* and *ФPSII* of maize seedlings under Cd stress, with more obvious performance in Fenghe 6. Studies have shown that Spd can stimulate ATP synthesis, induce the accumulation of thylakoids, and increase the rate of linear electron flow (LEF) and the photochemical efficiency of PSII (Li et al., 2018). Therefore, Hemin under Cd stress can increase the PSII photochemical efficiency of plants and improve plant growth and development. Non-photochemical quenching plays an important role in regulating the distribution of light energy. *NPQ* dissipates excitation energy in the form of heat through the lutein cycle (Wang et al., 2014). In our study, Cd stress significantly increased leaf *NPQ*, and *NPQ* of Tiannong 9 was higher than that of Fenghe 6, indicating that Fenghe 6 can effectively dissipate excess light energy, thereby reducing the damage to photosynthetic organs. Hemin can improve the non-photochemical quenching level of maize seedlings under Cd stress, increase energy dissipation, and protect chloroplasts from solid light.

The internal mechanism of the effect of Cd on photosynthesis is still unclear. Some studies believe that Cd affects the photolysis system through manganese protein to cause a decline in photosynthesis, and Cd^2+^ causes part of the LHCII depolymerization. Other studies believe that Cd affects photosynthesis by inhibiting the reaction steps in the Calvin cycle (Baryla et al., 2001). Previous studies have shown that the CO_2_ exchange rate, stomatal conductance, and transpiration rate of pigeon pea are reduced under Cd stress, and photosynthetic organelles (especially LHCII) are damaged, which is a comprehensive influence on the photosynthesis of plants through a variety of factors (Liu and Moriguchi, 2007). Plants’ uptake of heavy metal ions leads to conformational changes in enzymes related to the chlorophyll synthesis pathway, resulting in reduced enzyme activity and leaf discoloration (Maiti et al., 2012). Our study showed that *P_n_*, *g_s_* and *T_r_* of leaves after Hemin+Cd treatment increased, *qP* value of the fluorescence parameter, *F_v_/F_m_*, *ETR* increased significantly, which was conducive to improving PSII photosynthetic system. Hemin promoted the elevation of leaf photosynthetic pigment content under Cd stress, improved the ratio of *Chla* and *Chlb* parameters, elevated activities of RUBPCase and PEPCase, increased total lutein cycle pool (VAZ) value, and significantly enhanced DEPS de-epoxidation, which effectively alleviated the effects of Cd stress on leaf photosynthesis stomatal restriction and non-stomatal restriction.

Plant cells’ production and elimination of free radicals and reactive oxygen species are in a dynamic balance. TBARS is the peroxidation product of cell membrane lipid, and its content can reflect the degree of membrane lipid peroxidation (Jiao et al., 2018). The results showed that the TBARS content in maize tissues increased significantly after Cd treatment. Hemin effectively reduced the content of membrane lipid peroxidation TBARS in leaf tissues, reduced the degree of membrane lipid peroxidation, and ensured the integrity of cell membranes under Cd stress. In our study, Cd stress induced ROS production, increased O_2_^−^· production rate and H_2_O_2_ content. Moreover, it increased MDA content level, triggered membrane lipid peroxidation, and resulted in membrane structure and function damage, which was more severe in Fenghe 6 than in Tiannong 9. With the extension of Cd stress time, the MDA content of the two varieties of maize seedling leaves continued to increase. Hemin reduced the membrane lipid peroxidation induced by Cd stress, reduced the number of stained spots, ROS content, and EL, enhanced the enzyme and non-enzyme components of the antioxidant system, with a more significant impact on Fenghe 6. Compared with Cd treatment, the MDA content in Tiannong 9 and Fenghe 6 after Hemin+Cd treatment decreased, indicating that Hemin significantly reduced the MDA content and relative conductivity of the seedlings, which alleviated the cell membrane damage caused by Cd stress.

The aggravation of Cd stress increases the degree of membrane lipid peroxidation, which was regulated by plants’ enzymatic and non-enzymatic antioxidant systems (Gajewska and Sklodowska, 2010). In our study, SOD activity of maize seedlings increased significantly under Cd stress. After 1–2 days of Cd stress, CAT activity in Fenghe 6 decreased, indicating that the CAT scavenging effect on H_2_O_2_ of sensitive varieties decreased under Cd stress, and the production rate of O_2_^−^· and the content of H_2_O_2_ and MDA continued to increase. However, Hemin enhanced SOD and CAT activities under Cd stress, suggesting that Hemin could regulate the enzyme activity of the antioxidant system and eliminate excess ROS induced by Cd stress. Previous studies have shown that chloroplasts and mitochondria are the primary sites for producing active oxygen species in plants (Ding et al., 2014), and exogenous substances can directly act on the respiratory chain of mitochondria to affect the production of reactive oxygen species (Srivastava et al., 2014). In our study, Hemin can reduce the rate of O_2_^−^· production and the content of H_2_O_2_, which may be due to the reduction of active oxygen species production by the electron transfer rate.

Active oxygen generated by Cd through enzymatic and non-enzymatic reactions can cause damage (Fojtová and Kovařík, 2000), resulting in membrane damage and EL. In our study, Cd stress reduced the AsA content of maize. APX and AsA can reduce H_2_O_2_ to H_2_O. In our study, the increase of APX activity under Cd stress was responsible for the decrease of AsA, which is consistent with the results of previous studies (Anjum et al., 2016). Furthermore, Hemin increased the activity of MDHAR and DHAR, which may increase the AsA content and reduce the DHA content under Cd stress. The non-enzymatic antioxidant GSH oxidized to GSSG after participating in the removal of H_2_O_2_. After Cd treatment, plants poison soybean nodules and accelerate their senescence through oxidative stress (Song et al., 2009). Cadmium can cause the change of functional membrane areas by inducing a lipid peroxidation reaction. Our study showed that the content of GSH and GSSG increased with the duration of Cd stress. While GSH content in leaves increased and GSSG content decreased after Hemin treatment under Cd stress, and GR can reduce GSSG to GSH, which was very important to maintain reduced GSH. In our study, Cd stress increased the GR activity of maize seedlings, and Hemin further enhanced its activity. The ratio of AsA/DHA and GSH/GSSG can indicate the dynamic changes of cell redox state (Wang et al., 2007, 2011). Our study showed that exogenous Hemin increased the ratio of AsA/DHA and GSH/GSSG in maize under Cd stress, indicating that Hemin effectively increased the activity of the ROS system, thereby further reducing the rate of O_2_^−^· production, H_2_O_2_ and MDA content, and alleviating oxidative damage caused by Cd stress. In our study, Cd stress reduced the activity of enzyme antioxidants and non-enzymatic antioxidants in above-ground and roots, indicating that Cd stress destroyed the antioxidant enzyme system in plants.

Polyamine accumulation and metabolism changed considerably in maize under adversity conditions (Alcázar et al., 2011). Polyamine content decreases with increasing stress, while promoting the ADC pathway and increasing the Put content. SAMDC gene expression can increase the polyamine content, and DAO and PAO are polyamines degrading enzymes associated with plant stress resistance (Ahsan et al., 2007). Previous studies have shown that the use of exogenous substances can increase the polyamine content in maize plants under adversity, which is beneficial in reducing the inhibition of electron transfer and photosynthetic phosphorylation caused by the excessive acidification of the chloroplast inner cavity, which has a positive effect on improving plant stress resistance (Kramer et al., 2004). Our study showed that the Put content increased after Cd and Hemin+Cd treatment, significantly higher than CK and Hemin. Tiannong 9 accumulated more endogenous free polyamine than Fenghe 6 in response to stress. The content of Put, Spd and Spm increased after Hemin treatment. In our study, Cd stress significantly increased the free polyamines (Put, Spd, and Spm) content in maize seedlings, and the polyamines content in Tiannong 9 was higher than that in Fenghe 6.

Previous studies have shown that exogenous substance application can increase Spd and Spm content in the bound state under adversity (Li et al., 2018). Our study showed that the conjugated polyamine content of the two maize seedlings from the 1st to the 4th day after Cd treatment showed a gradual increase and reached the maximum at the 4th day compared with CK. Studies have shown that increasing bound Put content enhances thylakoid membranes’ stability, thereby enhancing tobacco protection from high light stress (Ioannidis et al., 2012). External stress may induce the expression of ADC2, SPDS1, and SPMS genes by increasing the ABA content (Alcázar et al., 2011). ADC, ODC and SAMDC are the key enzymes for synthesizing polyamines. DAO and PAO catalyze the decomposition of polyamines. Our study showed that Cd stress significantly increased the activities of ODC and SAMDC in the leaves of the two varieties of maize seedlings, and Hemin enhanced the increase in ODC activity induced by Cd stress. We suggested that Cd stress altered the level and form of polyamines accumulation by affecting the activities of polyamine enzymes in maize, and also indicated that the ability of maize seedlings to tolerate Cd stress was related to the ability of seedlings to maintain high ADC, SAMDC, ODC, and DAO enzyme activities and low PAO enzyme activities in response to Cd stress. Our study showed that Hemin enhanced the tolerance of maize under Cd stress by changing the polyamines level of plants, which was beneficial in reducing the oxidative damage of plants.

## Conclusion

**Figure 10:**
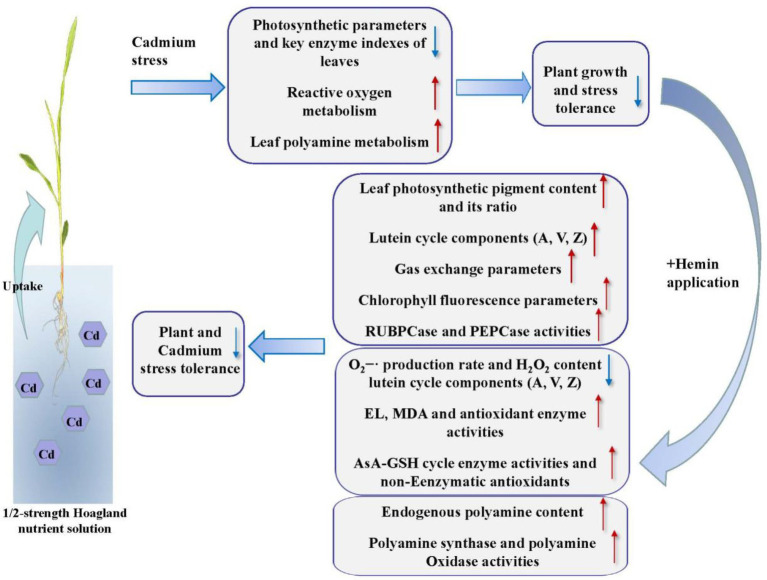
The physiological and biochemical response models of leaf photosynthesis, AsA-GSH cycle and polyamine metabolism enhance by Hemin under Cd stress. The red arrow (↑) indicates promoting effect and the blue arrow (↓) indicates inhibiting effect.

In conclusion, our results showed that Hemin can enhance the Cd stress tolerance of maize. Hemin increased maize photosynthetic pigment content, induced the activity increase of photosynthetic enzymes, significantly increased leaf total lutein circulation library, and strengthened de-epoxidation of lutein cycle, thus improving leaf photosynthesis and chlorophyll fluorescence parameters under Cd stress. At the same time, Hemin elevated the reactive oxygen metabolism capacity and the balance of antioxidant system in maize leaves and reduced the degree of membrane lipid peroxidation, thus maintaining the integrity of cell membrane structure and function under Cd stress. In addition, Hemin promoted the resistance to Cd stress ability by regulating the content of polyamine and enzyme activities in leaves under Cd stress. This study can provide the theoretical and experimental basis for Hemin to be applied to maize stress resistance production, significantly to improve the ability of maize to resist Cd stress at the seedling stage (Figure 10).

## Data availability statement

The original contributions presented in the study are included in the article/Supplementary material; further inquiries can be directed to the corresponding authors.

## Author contributions

LP, YW, and XL collected the samples, analyzed the samples, and wrote the manuscript. GS, SZ, JY, YC, and YM made a contribution to software and data curation of the manuscript. ML and WG made a contribution on design of the work, analysis, and revised the manuscript. All authors contributed to the article and approved the submitted version.

## Funding

This research was funded by the Postdoctoral Science Foundation of China (2018 M631905), the National Natural Science Foundation of Heilongjiang Province, China (LH2020C096), Heilongjiang Academy of Agricultural Sciences, Application Research and Development Projects (2020YYYF028), and the Innovation Spanning Project of Agricultural Technology (HNK2019CX12–12).

## Conflict of interest

YC was employed by the company Heilongjiang Kenfeng Seed Industry Co., Ltd.

The remaining authors declare that the research was conducted in the absence of any commercial or financial relationships that could be construed as a potential conflict of interest.

## Publisher’s note

All claims expressed in this article are solely those of the authors and do not necessarily represent those of their affiliated organizations, or those of the publisher, the editors and the reviewers. Any product that may be evaluated in this article, or claim that may be made by its manufacturer, is not guaranteed or endorsed by the publisher.
